# Editorial: Advanced technologies in bioengineering to fight antimicrobial resistance

**DOI:** 10.3389/fbioe.2023.1182463

**Published:** 2023-03-27

**Authors:** R. Vázquez, V. Rivero-Buceta, R. del Campo, I. Poblete-Castro, C. Herencias

**Affiliations:** ^1^ Department of Biotechnology, Faculty of Bioscience Engineering, Ghent University, Ghent, Belgium; ^2^ Centro de Investigaciones Biológicas Margarita Salas, Madrid, Spain; ^3^ Servicio de Microbiología, Hospital Universitario Ramón y Cajal and Instituto Ramón y Cajal de Investigación Sanitaria (IRYCIS), Madrid, Spain; ^4^ Centro de Investigación Biomédica en Red de Enfermedades Infecciosas-CIBERINFEC, Instituto de Salud Carlos III, Madrid, Spain; ^5^ Biosystems Engineering Laboratory, Department of Chemical and Bioprocess Engineering, Universidad de Santiago de Chile (USACH), Santiago, Chile

**Keywords:** antimicrobial resistance, phage therapy, gene editing systems, microbial growth, natural toxin production

## Introduction

The rise in antibiotic resistance has become a global health crisis, claiming millions of lives annually (Murray et al., 2022). It is essential to counter this threat by developing new therapeutic regimes that address the treatment of antibiotic-resistant bacteria and the reduction in the spread of resistance genes or high-risk clones carrying these genetic mechanisms. The fight against antibiotic resistance is becoming an increasingly interdisciplinary effort, involving not only the discovery and synthesis of new drugs and treatments, such as antimicrobial peptides, phage therapy and phage endolysins (Lin et al., 2017), and gene editing with CRISPR/Cas systems (Bikard et al., 2014) but also the integration of these treatments into wider healthcare programs. Microbiological and evolutionary insights are also crucial for informing clinical interventions (e.g., optimization of microbial growth conditions and the use of collateral sensitivity Herencias et al., 2021). Success in this struggle against antibiotic resistance requires close collaboration among scientists, engineers, and healthcare practitioners.

This Research Topic includes six research contributions highlighting the diversity and latest innovations in microbiology and biotechnology, covering areas ranging from optimizing microbial growth to engineering innovative solutions for infectious diseases.

## Phage therapy

Bacteriophages (or simply phages) are viruses that infect bacterial populations, including clinical pathogens of human or animal origin. Phages are superbly adapted antimicrobial agents that are an emerging alternative to classical antibiotics for treating bacterial infections (Lin et al., 2017). However, the typically narrow host range specificity of phages and the limited understanding of the pathogen-phage dynamic behavior *in vivo*, among other Research Topic, still hamper their use (Vázquez et al., 2022). To improve the efficacy and reproducibility of phage therapy, the host range of well-characterized lytic phages (e.g., T4 and T7) needs to be modified by swapping their receptor-binding proteins (RBPs). In one study, the RBP of the temperate phage ΦNP was identified as ORF6 and was shown to belong to a family of over 200 members, including phages targeting several human pathogens (*Klebsiella pneumoniae*, *Escherichia coli* O157:H7, *Salmonella* spp, and *Shigella* spp.) (Magaziner and Salmond).

In a separate study, the protein PtsHN10M was identified and shown to function as an RBP against *C. difficile* (Phetruen et al.). This protein was found to interact with the outer surface of *Clostridioides difficile* cells *via* the surface-layer protein A, highlighting the potential of PtsHN10M as a part of phage engineering and phage therapy against *C. difficile* infections. This approach could be a new strategy for countering this pathogen while avoiding the risks of fecal microbiota transplantation, which is currently the most effective procedure for recurrent *C. difficile* infections.

The bacteriophage KL-2146 infects NDM-1-carrying *K. pneumoniae* BAA-2146, which has been characterized in another study (Gilcrease et al.). KL-2146 belongs to the Drexlerviridae family and has a reversible host specificity after the re-infection of its original host. In addition, the authors demonstrated that, under biofilm conditions, KL-2146 can infect both antibiotic-resistant and antibiotic-sensitive strains.

## Gene editing systems

Developing gene editing systems has greatly improved the ability to study genetic diseases and develop new treatments. Several gene editing systems are available, including CRISPR-Cas9, which works by using specific nuclease activity at specific locations, allowing for the insertion, deletion, and alteration of genetic material (Bikard et al., 2014). Thus, studying the activity of various protein effectors (nucleases) will be crucial for success in its applications in areas such as disease diagnosis, prevention, and treatment. For example, Zhang et al. compared the nuclease activity of three effectors (Nme1Cas9, LwaCas13a, and RfxCas13d) to regulate gene expression in *S. cerevisiae* (Zhang et al.). The authors reported that the Nme1Cas9 variant could be used as an RNase *in vivo,* whereas the LwaCas13a and RfxCas13d variants appeared to be non-functional, highlighting the challenges associated with using Cas13 proteins in *Saccharomyces cerevisiae*.

## Optimization of microbial growth and natural toxin production

Understanding the virulence and physiology of pathogens will be crucial for countering antibiotic resistance. *Pseudomonas aeruginosa* is an opportunistic pathogen that can cause infections in individuals with weakened immune systems, such as those with cystic fibrosis, immunocompromised patients, and burn victims (Fernández-Olmos et al., 2013). Pyocyanin production by *Pseudomonas aeruginosa* has been linked to the persistence of the bacterium in these infections, as well as the development of antibiotic resistance (Meirelles et al., 2021). As a result, understanding the mechanisms involved in pyocyanin production and how to control it has become an important area of research in the field of medical microbiology. For example, Jabłońska et al. studied the effects of electromagnetic fields (EMFs) on pyocyanin production by *P. aeruginosa* (Jabłońska et al.), aiming to assess the impact of the exposure time and culture volume on the microorganism’s growth rate, respiration, and pyocyanin production. The results showed that exposure to EMFs slightly stimulated the growth of *P. aeruginosa* and significantly increased pyocyanin production. Thus, different types of EMFs, frequency, exposure time, and volume can be used interchangeably to achieve different bioprocess goals.

Another area of interest is the use of mathematical models to optimize fermentation processes to propagate beneficial bacteria, such as *Lactobacillus* species. Dulf et al. studied the metabolism and interaction patterns of *Lactobacillus plantarum* and *Lactobacillus casei* under varying conditions to increase bread quality by releasing bioactive compounds when soy flour is added to wheat flour (Dulf et al.). The authors used mathematical modeling to compute multiple regression and artificial neural networks to optimize the fermentation process by following the growth kinetics and viability of the lactic acid-bacterial strains. These models yielded optimized bacterial growth, improving microbial effectiveness as probiotics or bio-preservatives.

Overall, the field of bioengineering is making significant progress in the struggle against antimicrobial resistance. By developing innovative technologies and therapies, scientists from various fields are working together to ensure that future generations will have access to effective treatments for bacterial infections.
